# Plenary 5 Sustaining high quality care: interprofessional training for our clinical and public health workforce

**DOI:** 10.1093/eurpub/ckac128.005

**Published:** 2022-10-25

**Authors:** Organised by: ASPHER, EHMA, Moderators: John Middleton (ASPHER), Tara Chen (ASPHER)

## Abstract

The plenary session will align with the main conference theme ‘Strengthening health systems: improving population health and being prepared for the unexpected’ and providing a balanced perspective on public health and healthcare interprofessional linkages for training of the two workforces integral for a well-functioning, responsive and robust health system.

Natasha Azzopardi-Muscat, WHO Regional Office for Europe who will set the scene for the first Keynote with the WHO Athens Office work on European Programme of Work (EPW) 2020–2025 – ‘United Action for Better Health in Europe’ reflecting on the coherence of policies, structures and resources for quality of health care and implications for policy dialogue, policy formulation and technical assistance at the regional, sub-regional and country levels. She will also speak to the WHO/EURO Regional HRH Report presented in September 2022 and the WHO-ASPHER Roadmap to Professionalization launched in February 2022 offering pragmatic recommendations for action to professionalize the public health workforce as a response to growing public health needs.

EHMA Director George Valiotis will follow with a Keynote speaking to the key EU agenda on skills for health, with the EHMA led BeWell (2022-2025) project: Investing in the upskilling and reskilling of the European health workforce. The multi-partner consortium aims to build a movement of healthcare stakeholders which support and contribute to the development, implementation, and upscaling of a strategy on the upskilling and reskilling of the European health workforce addressing the skills needed to support the digital and green transition within the health ecosystem enabling all health professionals to be better prepared to face future challenges and adapt to ever-evolving societal contexts.

Fatai Ogunlayi, UK Public Health Specialty Registrar will speak to the global level agenda with focus on the WHO Roadmap: “Building the Public Health and Emergency Workforce’. The roadmap is designed to provide countries with a differentiated and progressive approach to acknowledge varying capacities and contexts with provision of guidance and tools; and support progress to full implementation towards a strengthened public health workforce delivering all essential public health functions for universal health coverage, health security and improved health and wellbeing.

Laurent Chambaud, Dean, EHESP School of Public Health, will provide a country perspective with discussion of work toward official agreements on the One Health approach, which recognizes that the health of people is closely connected to the health of animals and our shared environment, and the interprofessional context and training required to achieve it.

**Keynote speaker:**

**Natasha Azzopardi Muscat**

WHO Europe

**George Valiotis**

European Health Management Association, Brussels, Belgium

**Speakers/Panellists:**

**Fatai Ogunlayi**

UK Public Health Specialty Registrar, UK

**Laurent Chambaud**

Andrija Stampar Medallist 2022, France

**Anett Ruszanov**

Director of Policy and Programmes, EHMA

